# ﻿*Melanasterasinica* He & Burckhardt, sp. nov., a new psylloid species (Hemiptera, Psylloidea, Liviidae) from China developing on *Grewia* sp. (Malvaceae)

**DOI:** 10.3897/zookeys.1204.123740

**Published:** 2024-06-06

**Authors:** Zhixin He, Daniel Burckhardt, Xinyu Luo, Rongzhen Xu, Wanzhi Cai, Fan Song

**Affiliations:** 1 Department of Entomology & MOA Key Lab of Pest Monitoring and Green Management, College of Plant Protection, China Agricultural University, Beijing, 100193, China China Agricultural University Beijing China; 2 Naturhistorisches Museum, Augustinergasse 2, 4001 Basel, Switzerland Naturhistorisches Museum Basel Switzerland

**Keywords:** Jumping plant lice, Liviinae, Oriental Region, Paurocephalini, Sternorrhyncha, taxonomy

## Abstract

*Melanasterasinica* He & Burckhardt, **sp. nov.**, a new psylloid species developing on *Grewia* sp., is described from Hainan, China. It is the first *Melanastera* species reported from Asia and China, and the second species from the Old World. While New World species of *Melanastera* are mostly associated with the plant families Melastomataceae and Annonaceae, the two Old World species develop on the malvaceous *Grewia*, a host otherwise used in psylloids by two *Haplaphalara* species. The new species is described, diagnosed and illustrated, and its host plant and biogeographic ranges are discussed.

## ﻿Introduction

Jumping plant lice or psylloids constitute the superfamily Psylloidea, with slightly over 4000 species worldwide (Burckhardt et al. 2023). They are characterised by narrow host ranges mostly associated with eudicots and magnoliids, while hosts from the monocots and conifers are rare. In addition, related psylloid species tend to develop on related hosts (Hodkinson and Bird 2000; Burckhardt 2005a, 2005b; Burckhardt et al. 2014; Ouvrard et al. 2015). Ouvrard et al. (2015) conducted an analysis of the host patterns of psylloids worldwide and found that psylloid hosts are not evenly distributed across angiosperms. Instead, they identified particular plant taxa that are preferred hosts for psylloids. The Malvaceae is such a family, ranking as the 9^th^ most important host taxon while it constitutes only the 12^th^ largest plant family worldwide. Apart from a single species of Triozidae, *Bactericeralavaterae* (Van Duzee, 1924) (Burckhardt and Lauterer 1997), Malvaceae hosts all the members of the subfamily Carsidarinae (Carsidaridae) (Hollis 1987) and many species of the tribe Paurocephalini (Liviidae, Liviinae) (Burckhardt et al. 2023). Of the Paurocephalini, all of the seven recognised genera include members associated with Malvaceae. All species of *Klyveria* and *Woldaia*, and many species of *Diclidophlebia*, *Haplaphalara* and *Liella* develop on Malvaceae, while only a few species of *Melanastera* and *Paurocephala* are associated with this family (Burckhardt et al. 2023; Serbina et al. in press).

Recently, an undescribed *Melanastera* species was discovered on the malvaceous genus *Grewia* in China. *Melanastera* currently comprises more than 60 extant species in the New World, many of which develop on Melastomataceae and Annonaceae (Burckhardt et al. 2023; Serbina et al. in press). In the Old World, the genus is represented only by a single Afrotropical species, also associated with *Grewia* (Burckhardt et al. 2023).

Here, we describe *Melanasterasinica* He & Burckhardt, sp. nov., which represents the first record of the genus from China and Asia. Morphological information and illustrations are provided for adults and fifth instar immatures.

## ﻿Material and methods

Material was examined from the Entomological Museum of the China Agricultural University, Beijing, China (**CAU**) and the Naturhistorisches Museum, Basel, Switzerland (**NHMB**).

The morphological terminology accords with Burckhardt et al. (2023) and Serbina et al. (in press). For examining morphological structures under the compound microscope, the whole insect was cleared in a hot potassium hydroxide (KOH) solution for ten minutes, washed in distilled water and then mounted on a slide in glycerine. Measurements were taken from slide-mounted specimens. Photos were taken with a Nikon SMZ18 microscope (Tokyo, Japan) attached to a Cannon 7D camera (Tokyo, Japan). Helicon Focus version 5.3 (Helicon Soft Ltd., Kharkiv, Ukraine) was used for image stacking. Line drawings were made using an Olympus BX41 microscope. Photoshop 2020 (Adobe Systems Inc., USA) was used to edit photos including adjustments of background colour and cropping without modifying any characters of specimens. The concept of host plants adopted here is that of Burckhardt et al. (2014). The nomenclature of plants accords with POWO (2024).

## ﻿Taxonomy

### ﻿ Melanastera sinica

Taxon classificationAnimaliaHemipteraLiviidae

He & Burckhardt sp. nov.

6B43D559-5AAB-5F7F-8550-F5DCB0BFCE7B

https://zoobank.org/6F961330-7600-4B39-9885-BBC1F01FF45F

Figs 1 , 2 , 3

#### Type locality.

China, Hainan: Ledong County, Jianfengling, Mingfenggu, 18°74'24"N, 108°84'81"E.

#### Type material.

***Holotype***: China • ♂; Hainan, Ledong County, Jianfengling, Mingfenggu; 18°74'24"N, 108°84'81"E; 24 Apr. 2016; X.-Y. Luo leg.; on Grewiacf.chuniana; CAU, dry mounted. ***Paratypes***: China • 2 ♂, 7 ♀, 12 immatures; same data as holotype; CAU, NHMB, dry and slide mounted, and in 95% ethanol.

#### Diagnosis.

Adult. Body yellowish brown with small dark brown dots; forewing with each a broad medial and subapical light brown band and small, dark brown irregular spots. Metatibia with 3+4 grouped apical metatibial spurs separated by five unsclerotised bristle-like setae anteriorly. Forewing oval, widest in apical third; pterostigma long, strongly widening to middle; surface spinules present in all cells, covering membrane up to the veins; irregularly spaced to form groups of 5–6 spinules. Male proctiger weakly expanded posteriorly. Paramere, in lateral view, subrectangular with antero-apical sclerotised tooth. Aedeagus two-segmented; distal segment lacking ventral process. Female proctiger with relatively straight dorsal margin; apex obliquely truncate. Circumanal ring cruciform. – Fifth instar immature. Antenna 10-segmented. Forewing pad with 5 marginal subacute sectasetae. Tarsal arolium narrowly lamellar, widening to apex which is rounded; about twice as long as claws. Caudal plate with anterior margin distant from anterior margin of extra pore fields; with 2 lateral sectasetae on either side near fore margin, and three pointed sectasetae on either side of circumanal ring dorsally.

#### Description.

**Figure 1.**
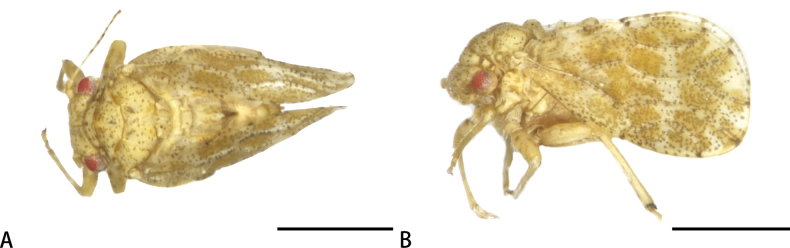
Habitus of adults of *Melanasterasinica* He & Burckhardt, sp. nov. **A** dorsal view **B** lateral view. Scale bars: 1.0 mm.

**Adult. *Colouration*.** Body (Fig. 1A, B) yellowish brown. Head and thorax covered with sparse small brown dots. Antenna yellow to yellowish brown, with apices of segments IV–IX and entire segment X dark brown to black. Femora with brown spots. Forewing yellowish with light brown pattern consisting of each a broad medial and subapical band and small, brown irregular spots, veins pale yellow.

**Figure 2.**
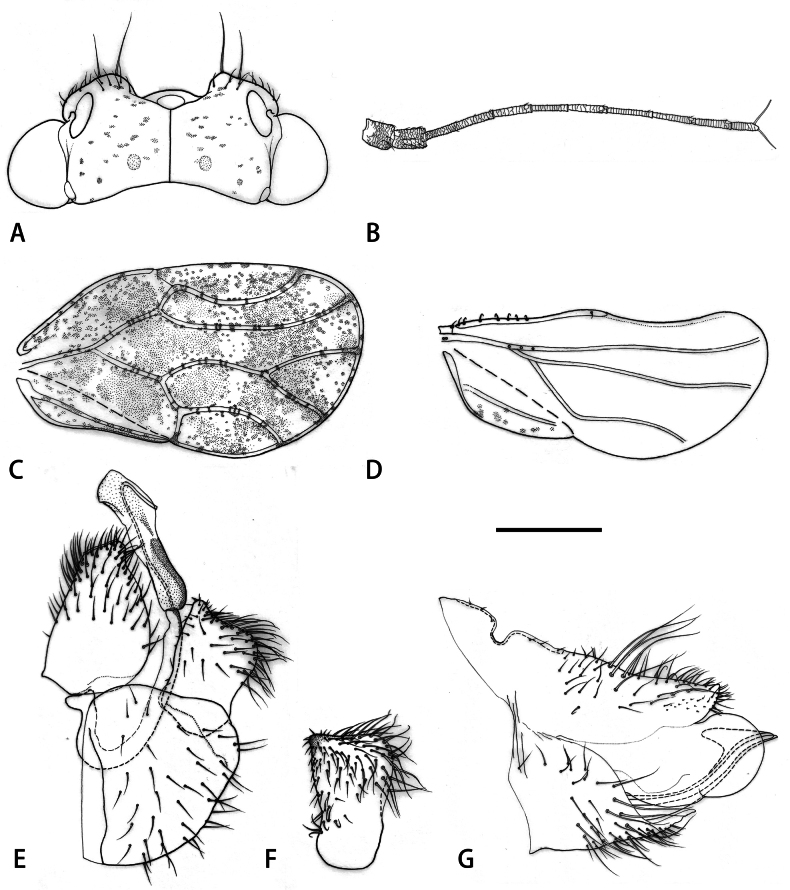
*Melanasterasinica* He & Burckhardt, sp. nov., adult **A** head **B** antenna **C** forewing **D** hindwing **E** male terminalia **F** inner face of paramere **G** female terminalia. Scale bars: 0.25 mm (**A**); 0.3 mm (**B**); 0.5 mm (**C**, **D**); 0.1 mm (**E**–**G**).

***Structure*.** Head, in lateral view (Fig. 1B), inclined at 45° from longitudinal body axis; in dorsal view (Fig. 1A), about as wide as mesoscutum. Vertex (Fig. 2A) subrectangular, about half as long as wide; surface with fine microsculpture and microscopical setae; median suture developed; posterior margin weakly concave. Genae weakly rounded, with each a pair of long setae on either side of frons (Fig. 2A). Frons relatively large, triangular. Eyes hemisphaerical. Antenna (Fig. 2B) 1.7–1.8 times as long as head width, with a single, large subapical rhinarium on each of segments IV, VI, VIII and IX; relative length of flagellar segments as 1.0: 0.5: 0.5: 0.5: 0.6: 0.6: 0.5: 0.4; relative length of segment 10 and terminal setae as 1.0: 1.3: 1.1. Clypeus flattened, in ventral view almost triangular. Thorax distinctly arched, with fine microsculpture; mesoscutellum swollen; metapostnotum with small subacute, laterally compressed tooth. Metacoxa with relatively short horn-shaped, blunt meracanthus; metatibia 1.0 times as long as head width, slender, weakly expanded apically; with 3+4 grouped apical metatibial spurs separated by five unsclerotised bristle-like setae anteriorly. Forewing (Fig. 2C) 2.7–3.1 times as long as head width, 1.6–1.8 times as long as wide, oval, widest in apical third; wing apex in the middle of cell r_2_; veins densely clothed in conspicuous setae; vein C+Sc straight in basal two thirds, strongly bent in apical third; pterostigma long, strongly widening to middle; vein Rs relatively straight in the middle, curved in a 30° angle to costal margin apically; vein M weakly, irregularly curved; veins M_1+2_ and M_3+4_ slightly shorter than M; vein Cu shorter than M+Cu; vein Cu_1a_ evenly curved; vein Cu_1b_ straight, slightly shorter than Cu; surface spinules present in all cells, covering membrane up to the veins, along veins slightly finer; irregularly spaced to form groups of 5–6 spinules. Hindwing (Fig. 2D) slightly shorter than forewing, with indistinctly grouped costal setae. Visible abdominal tergites III–V with a tubercular bump in the middle in both sexes.

***Terminalia*** (Fig. 2E–G). Male proctiger (Fig. 2E) 1.2–1.3 times as long as head width, broad with posterior margin produced; in lateral view, widest in basal third. Subgenital plate (Fig. 2E) subglobular, weakly irregularly curved dorsally. Paramere (Fig. 2E, F), in lateral view, subrectangular with antero-apical, partly sclerotised tooth-like process; posterior margin weakly sinuate; outer and inner face covered in long setae in apical half, denser on inner face and along apical and posterior margins. Aedeagus (Fig. 2E) two-segmented; distal segment, in lateral view, tubular and slightly angular postero-apically, lacking ventral process; sclerotised end tube of ductus ejaculatorius moderately long, relatively straight. – Female terminalia (Fig. 2G) cuneate, moderately long. Proctiger 0.4 times as long as head width; in lateral view, dorsal margin, distal to circumanal ring, almost straight; with a transverse row of long setae in the middle and a lateral longitudinal row of long setae on either side in apical third; apex, in lateral view, obliquely truncate, apex slightly upturned. Circumanal ring 0.4 times as long as proctiger; in dorsal view, cruciform. Subgenital plate 0.6 times as long as proctiger; irregularly narrowing to pointed apex, in lateral view; beset with long setae in apical two thirds.

***Measurements*** (in mm; 3 ♂, 2 ♀). Total body length measured from anterior margin of vertex to tip of folded forewing ♂ 2.31–2.52, ♀ 2.42–2.68; antennal length ♂ 1.23–1.31, ♀ 1.24–1.34; metatibia length ♂ 0.70–0.73, ♀ 0.72–0.74; forewing length ♂ 1.87–2.12, ♀ 1.96–2.26; proctiger length ♂ 0.15–0.16 ♀ 0.26–0.28; paramere length 0.12–0.13; length of distal segment of aedeagus 0.13–0.14.

**Figure 3.**
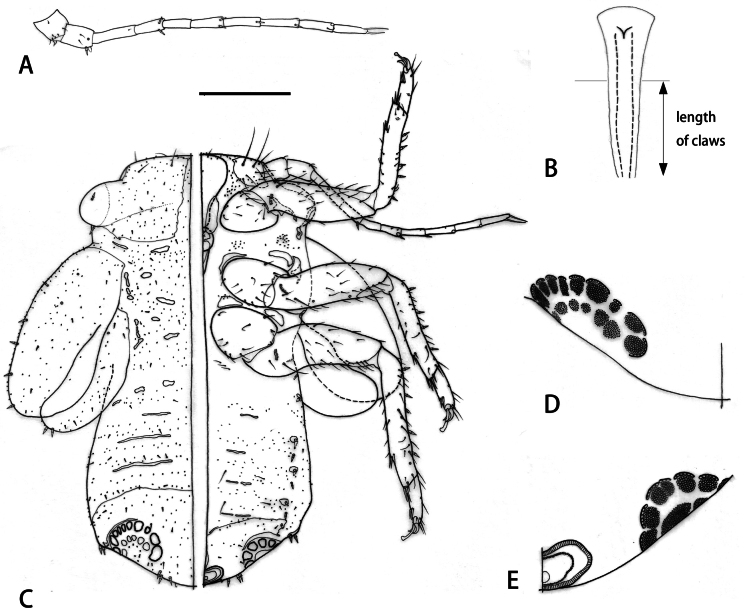
*Melanasterasinica* He & Burckhardt, sp. nov., fifth instar immature **A** antenna **B** tarsal arolium **C** habitus (left: dorsal side, right: ventral side) **D** detail of extra pore fields (dorsal side) **E** detail of extra pore fields and circumanal ring (ventral side). Scale bars: 0.15 mm (**A**); 0.03 mm (**B**); 0.2 mm (**C**); 0.01 mm (**D, E)**.

Fifth instar immature (Fig. 3). Body (Fig. 3C) 1.1–1.2 times as long as wide; sparsely covered in microscopic setae. Antenna (Fig. 3A) 10-segmented with a subapical rhinarium on each of segments IV, VI, VIII and IX, and following numbers of subacute sectasetae: 1 (0), 2 (2), 3 (0), 4 (2), 5 (0), 6 (2), 7 (1), 8 (1), 9 (0), 10 (0). Legs moderately long with 4–5 subacute sectasetae on tibiotarsi; tarsal arolium (Fig. 3B) narrowly lamellar, widening to apex which is rounded; about twice as long as claws. Forewing pad 0.7 times as long as antenna, bearing 5 moderately large marginal subacute sectasetae (Fig. 3C); hindwing pad with 2 marginal subacute sectasetae. Caudal plate (Fig. 3C–E) with anterior margin distant from outer band of extra pore fields, inner band of pores less distinct than outer band; with 2 sectasetae on either side laterally, and three pointed sectasetae on either side of circumanal ring dorsally.

Measurements (in mm; 2 immatures). Body length 0.98–1.01; antennal length 0.52–0.54; length of forewing pad 0.35–0.37; length of tarsal arolium 0.03–0.04; length of claws 0.01–0.02.

#### Etymology.

From the Latin adjective *sinicus* = Chinese, referring to the unexpected discovery of this mostly American genus in China.

#### Distribution.

China: Hainan.

#### Host plant.

Grewiacf.chuniana Burret (Malvaceae).

#### Comments.

Serbina et al. (in press) defined several species groups within *Melanastera* mostly on the basis of morphological characters of adults. *Melanasterasinica* is a member of the *curtisetosa*-group for the absence of a ventral process on the distal segment of the aedeagus. The *curtisetosa*-group is composed of four species from Brazil associated with Asteraceae (confirmed or likely hosts) and *M.pilosa* (Burckhardt et al. 2006) from Kenya and Tanzania, developing on *Grewiabicolor* Juss. (Malvaceae) (confirmed). *Melanasterasinica* differs from the four Brazilian species in the forewing with a much more expanded dark pattern, which is slightly expanding towards the apex (versus parallel-sided) and bears a broad (versus narrow) pterostigma, in the broad subrectangular paramere (versus narrow, lamellar), the 10-segmented antenna in the last instar immature (versus 9-segmented), and the host association with Malvaceae (versus Asteraceae). From the African *M.pilosa* with which it shares the expanded dark forewing pattern, the 10-segmented antenna in the last instar immature and the host genus *Grewia*, it differs in the broader paramere, the absence of long setae on the body and forewing, the broad (versus narrow) pterostigma, the broader paramere (versus narrower), and the apically broader and obliquely truncate (versus slender and subacute) female proctiger (Burckhardt et al. 2006).

## ﻿Discussion and conclusions

Burckhardt et al. (2023) suggested that, within Liviinae, new species are more likely to be discovered among the tropical Paurocephalini rather than among its sister group, the predominantly north-temperate Liviini. The discovery of *Melanasterasinica* in tropical Hainan supports this notion. *Melanastera* is primarily found in the Neotropical region (Serbina et al. in press), with only a single species known from the Old World so far, viz. *M.pilosa* (Burckhardt et al. 2006). *Melanasterasinica* represents the second species from the Old World and the first from Asia. The presence of an expanded dark pattern on the forewing in the adults and 10-segmented antennae in the last instar immatures, shared by the two Old World species, suggests a putative sister group relationship of *M.pilosa* and *M.sinica*. The two species also share the malvaceous *Grewia* as the host, representing a family absent among the hosts of the New World *Melanastera* species.

*Grewia* includes 277 species in Africa, Asia and Australia (POWO 2024). Among psylloids, another two species use *Grewia* as the host: *Haplaphalaragrewiae* (Kandasamy, 1986) and *H.menoni* (Mathur, 1975), two species of Paurocephalini from India (Burckhardt et al. 2023). Targeted surveys of *Grewia* species in China will show, if additional, currently unknown psylloids are associated with this genus.

## Supplementary Material

XML Treatment for Melanastera sinica

## ﻿Additional information

### Author ORCIDs

Zhixin He https://orcid.org/0000-0002-9148-8157

Daniel Burckhardt https://orcid.org/0000-0001-8368-5268

Xinyu Luo https://orcid.org/0009-0001-0412-353X

Rongzhen Xu https://orcid.org/0009-0003-1831-2328

Wanzhi Cai https://orcid.org/0000-0002-8620-0446

Fan Song https://orcid.org/0000-0002-2900-4174

### Data availability

All of the data that support the findings of this study are available in the main text.
